# Electrospinning Preparation, Structure, and Properties of Fe_3_O_4_/Tb(acac)_3_phen/Polystyrene Bifunctional Microfibers

**DOI:** 10.3390/ma16124409

**Published:** 2023-06-15

**Authors:** Lina Liu, Ruifei Qin, Xiaofeng Fan, Kexin Wang, Xiujie Wang, Hao Wang, Yongjun Chen, Jintao Wang, Yi Wang

**Affiliations:** 1 Department of Mathematics and Physics, Luoyang Institute of Science and Technology, Luoyang 471023, China; 2 School of Environmental Engineering and Chemistry, Luoyang Institute of Science and Technology, Luoyang 471023, China

**Keywords:** electrospinning, Fe_3_O_4_, Tb(acac)_3_phen, microfiber, polystyrene

## Abstract

Compared to single functional materials, multifunctional materials with magnetism and luminescence are more attractive and promising; Thus, it has become an important subject. In our work, bifunctional Fe_3_O_4_/Tb(acac)_3_phen/polystyrene) microfibers with magnetic and luminescent properties (acac: acetylacetone, phen: 1,10-phenanthroline) were synthesized by simple electrospinning process. The doping of Fe_3_O_4_ and Tb(acac)_3_phen made the fiber diameter larger. The surface of pure polystyrene microfibers and microfibers doped only with Fe_3_O_4_ nanoparticles were chapped similar to bark, whereas the surface of the microfibers was smoother after doping with Tb(acac)_3_phen complexes. The luminescent properties of the composite microfibers were systematically studied in contrast to pure Tb(acac)_3_phen complexes, including excitation and emission spectra, fluorescence dynamics, and the temperature dependence of intensity. Compared with the pure complexes, the thermal activation energy and thermal stability of composite microfiber was significantly improved, and the luminescence of the unit mass of Tb(acac)_3_phen complexes in composite microfibers was stronger than that in pure Tb(acac)_3_phen complexes. The magnetic properties of the composite microfibers were also investigated using hysteresis loops, and an interesting experimental phenomenon was found that the saturation magnetization of the composite microfibers gradually increased with the increase in the doping proportion of terbium complexes.

## 1. Introduction

With the increasing demand for integrated devices, it is difficult for single-function materials to meet the needs of modern science and technology. Multifunctional materials have two or more functions. Therefore, Scientists are increasingly interested in multifunctional composite materials, owing to their wider application fields, such as nanosensors [1], drug delivery systems [2], biomedical engineering [3], and catalytic synthesis [4]. Luminescent–magnetic multifunctional materials have attracted wide attention, owing to their important applications in the medicine and biological fields [5,6,7]. Ghazimoradi et al. synthesized multifunctional core/double shells CoFe_2_O_4_/mSiO_2_-NH_2_/Poly(MAA-co-IA)/MF_3_ for the co-delivery of anticancer drugs (MTX and DOX) for breast cancer treatment [8]. Swain et al. synthesized multifunctional Fe_3_O_4_@BaMoO_4_:Eu^3+^ and used it for drug carrier [9]. Müssig et al. synthesized colorful luminescent magnetic supraparticles [10]. 

Rare earth luminescent materials exhibit unique chemical, electronic, and optical properties due to the f-f electron transition. Therefore, rare-earth luminescent materials have extensive applications on luminescence devices, displays, biolabeling, optical imaging photo-therapy, optical windows, miRNA detection [11,12,13], and so on. Magnetic nanomaterials have attracted increasing interest from scientists due to their potential applications, such as biomacromolecule separation, drug delivery and release, and MRI [14,15,16]. Among the magnetic materials, Fe_3_O_4_ has the advantages of high saturation magnetization, non-toxicity, good biocompatibility, and has a wide range of applications in the biomedical field [17,18,19]. Therefore, rare-earth luminescent materials and Fe_3_O_4_ nanoparticles have been employed as luminescent and magnetic materials in luminescent–magnetic composites [20,21].

**Figure 1:**
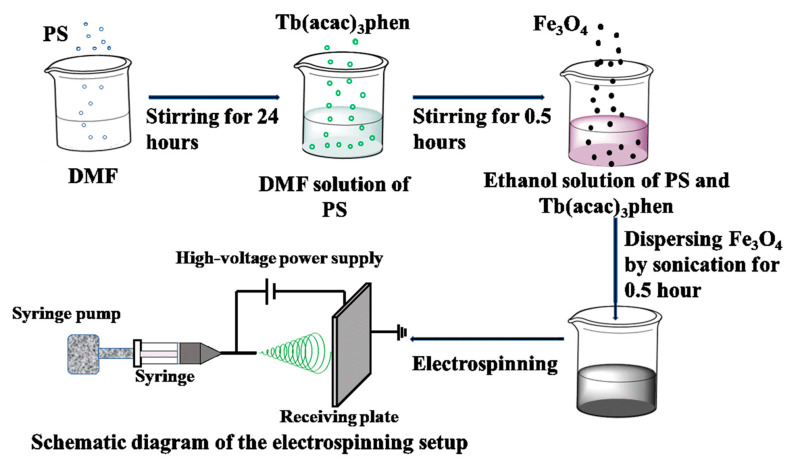
Schematic diagram of the synthetic procedure. Lina Liu et al.

Electrospinning is a relatively simple, straightforward, and versatile fiber-forming technology [22,23], which provides a unique way to fabricate continuous fibers or belts with 1D nanostructure [24,25]. This method shows an extensive application in filtration materials, optical fiber humidity sensors, electrode materials of Li-ion batteries, regenerative biology, cosmetics, skin regeneration, battery, filters, light-emitting diodes [20,26,27,28], and so on. In previous work, some groups have successfully fabricated luminescent–magnetic nanofibers [26,29,30] using the electrospinning method. Most of them are Janus structure fibers [20,24,26]. As far as we know, there is no report on the composite fibers of terbium complexes and Fe_3_O_4_ nanoparticles. Based on the above analysis, in this work, Fe_3_O_4_/Tb(acac)_3_phen/PS (polystyrene) multifunctional microfibers were synthesized via the single needle electrospinning process. The synthesis procedure of the multifunctional microfibers is shown in Figure 1. The structure and photoluminescent and magnetic properties of the multifunctional microfibers were investigated. This new kind of microfiber combines the advantages of rare-earth complexes and Fe_3_O_4_ NPs. The high-performance luminescent–magnetic bifunctional microfibers have potential applications in biomedical and biochemical fields.

## 2. Experimental Section

### 2.1. Materials

*N*,*N*-Dimethylformamide (DMF), anhydrous ethanol, ferric chloride hexahydrate (FeCl_3_·6H_2_O), ethylene glycol, anhydrous sodium acetate (NaAc), and ethylenediamine were purchased from Sinopharm Chemical Reagent Co., Ltd. (Shanghai, China). PS (molecular weight about 260,000), 1,10-phenanthroline (phen), Tetrabutylammonium chloride, acetyl acetone (acac), and terbium chloride hexahydrate (TbCl_3_·6H_2_O) were purchased from J&K Scientific (Beijing, China). The reagents used were all analytically pure and were not further purified before use. All chemicals were of analytical grade and were used as received without further purification.

### 2.2. Electrospinning of Fe_3_O_4_/Tb(acac)_3_phen/PS Microfibers

Tb(acac)_3_phen complexes and Fe_3_O_4_ nanoparticles were synthesized according to the traditional method [31,32]. In the preparation of precursor solutions, *N*,*N*-dimethylformamide (DMF) was used as the solvent. In a typical synthesis, the electrospinning solution was prepared by dissolving 0.3 mg/mL PS in DMF. To prepare a uniform fiber by electrospinning, 0.015 g tetrabutylammonium chloride was added under vigorous stirring, and then the precursor was stirred for 24 h. A certain amount of Tb(acac)_3_phen complexes was dissolved in that solution under magnetic stirring for 10 min. The mass ratios of Tb(acac)_3_phen complexes to PS for various samples were 20%, 30%, 40%, 50%, respectively. A series of Fe_3_O_4_/Tb(acac)_3_phen/PS precursor solutions with different Tb(acac)_3_phen complex contents were prepared by dispersing 0.1 g Fe_3_O_4_ nanoparticles into the solution using ultrasonic method.

Functional microfibers were prepared by the electrospinning method. The electrospinning process was carried out at room temperature and relative humidity, similar to previous literatures [29,33]. The syringe was placed on a syringe pump and used at a flow rate of 0.6 mL/h^−1^. The voltage was 15 kV, and the distance between the collecting plate and the needle was 25 cm. The bifunctional microfibers with Tb(acac)_3_phen accounting for 20%, 30%, 40%, and 50% of PS mass were labeled as samples 20Fe20Tb, 20Fe30Tb, 20Fe40Tb, and 20Fe50Tb, respectively. For comparison, we prepared pure PS microfibers, microfibers containing only 20% Fe_3_O_4_ nanoparticles, and microfibers containing only 40% Tb(acac)_3_phen complexes. The three samples were labeled as PS, 20Fe0Tb, and 0Fe40Tb, respectively. The mass ratios of Tb(acac)_3_phen complexes and Fe_3_O_4_ nanoparticles to PS in different samples are shown in Table 1.

### 2.3. Characterization

The phase structure of Fe_3_O_4_ nanoparticles and the bifunctional microfibers was performed by X-ray diffraction (XRD) (D8 ADVANCE, Cu Kα, λ = 1.5418 Å, Bruker, Karlsruhe, Germany). The SEM (scanning electron microscope) images and EDAX (energy spectrum) was measured on a Hitachi SU8020 microscope (Hitachi, Ltd., Tokyo, Japan, 3 KV). TEM (transmission electron microscope) was performed using a FEI Tecnai G2 F30 S-TWIN electron microscope (FEI, Inc., Valley City, ND, USA, 300 KV). The IR absorption spectra were measured in the range 400–4000 cm^−1^ using an FT-IR (Fourier transform infrared) spectrophotometer (Nicolet Niccolet IS10, Thermo Scientific™, Waltham, MA, USA). The fluorescence dynamics, dependence of emission intensity on temperature (100 K, 150 K, 200 K, 250 K, 300 K, and 350 K), and excitation and emission spectra were recorded using an Edinburgh FLS1000 spectrophotometer (Edinburgh Instruments Ltd., Livingstone, UK). The step was 1 nm, and the power of xenon lamp was 300 W. The conductivity data were measured on ST2643 ultra high resistance tester (Suzhou Jingge Electronic Co., Ltd., Suzhou, China) and the test voltage was 100 V. The hysteresis loops of samples (room temperature) were measured on a vibrating sample magnetometer (MPMS-3) manufactured by Quantum Design, Inc. (San Diego, CA, USA), and the range of magnetic field was −30,000 Oe to 30,000 Oe.

## 3. Results and Discussion

### 3.1. Structure and Morphology of Bifunctional Fe_3_O_4_/Tb(acac)_3_phen/PS Microfibers

**Figure 2:**
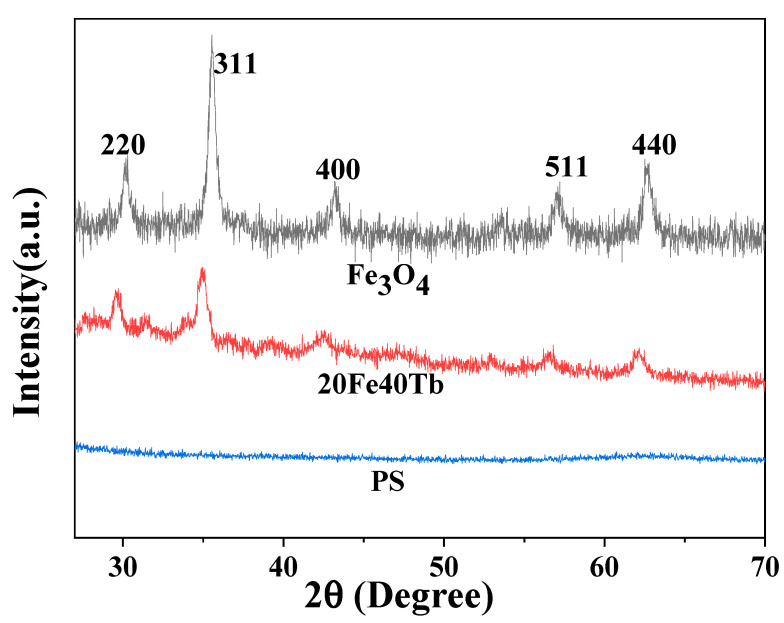
XRD patterns of Fe_3_O_4_ nanoparticles, sample 20Fe40Tb, and PS microfibers. Lina Liu et al.

The XRD patterns of sample 20Fe40Tb, Fe_3_O_4_ nanoparticles, and PS microfibers are shown in Figure 2. All diffraction peaks for Fe_3_O_4_ nanoparticles could be readily indexed to the face-centered cubic phase of Fe_3_O_4_, according to the Joint Committee on Powder Diffraction Standards (JCPDS) file on JCPDS 89-0688 [34]. The diffraction peaks of Fe_3_O_4_ were observed in sample 20Fe40Tb, which indicated that Fe_3_O_4_ nanoparticles were doped into the composite microfibers. The XRD pattern of PS microfibers showed no diffraction peaks, indicating that the PS microfibers were amorphous [29].

**Figure 3:**
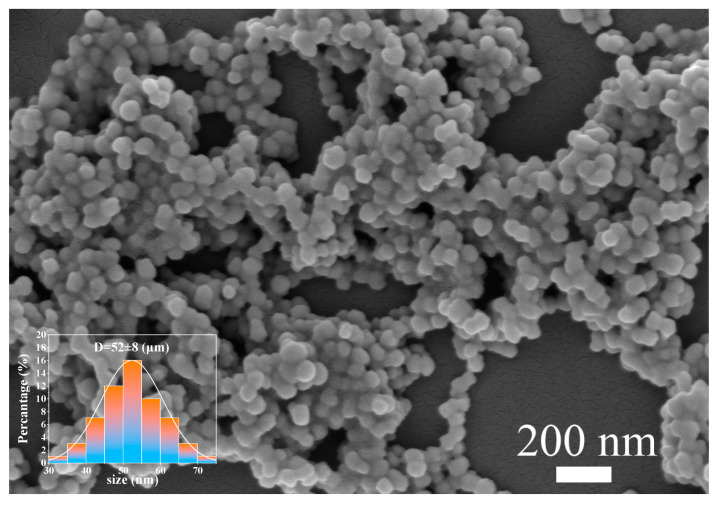
SEM image (inset: diameter distribution) of Fe_3_O_4_ nanoparticles. Lina Liu et al.

**Figure 4:**
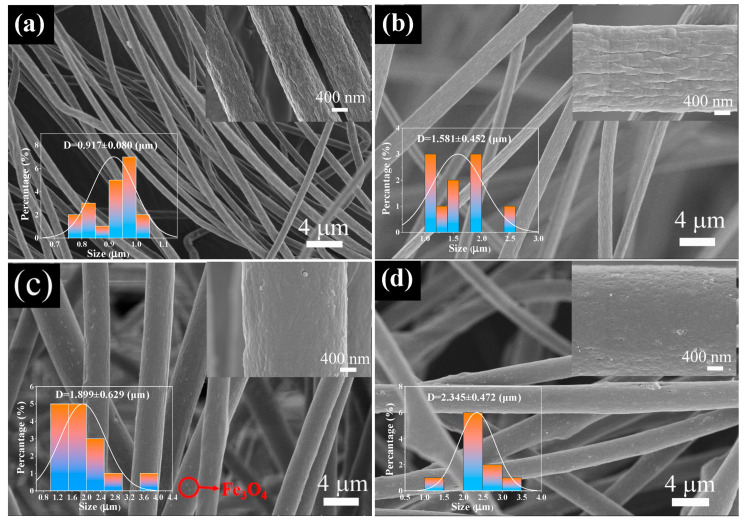
SEM images (inset: magnified view and diameter distribution) of (**a**) pure PS fibers; (**b**) sample 20Fe0Tb; (**c**) sample20Fe40Tb; (**d**) sample 20Fe50Tb. Lina Liu et al.

The SEM image of Fe_3_O_4_ nanoparticles are shown in Figure 3. The average diameter of Fe_3_O_4_ nanoparticles was estimated to be 52 nm. Figure 4 shows the SEM images of pure PS microfibers and samples 20Fe0Tb, 20Fe40Tb, and 20Fe50Tb. As can be seen from the figure, the diameters of pure PS microfibers were uniformly distributed, with an average diameter of about 0.9 µm. The average diameters of samples 20Fe0Tb, 20Fe40Tb, and 20Fe50Tb were about 1.6, 1.9, and 2.3 µm, respectively. The results indicated that the doping of Fe_3_O_4_ and Tb(acac)_3_phen made fiber diameters larger. It can be seen from the high-resolution SEM images of microfibers that the surfaces of pure PS fibers and fibers doped only with Fe_3_O_4_ nanoparticles were chapped similar to bark. The fiber surface became smooth, and the cracking phenomenon was weakened after doping with Tb(acac)_3_phen complexes. The electrospinning precursor solution conductivity values of the pure PS fibers and sample 0Fe40Tb were 4.00 × 10^−6^ and 1.23 × 10^−7^ S/cm, indicating that the doping of Tb(acac)_3_phen complexes reduced the conductivity of the electrospinning precursor solution, which influenced the fibers’ diameters and the fibers’ surface smoothness properties. As can be seen from Figure 4c,d, there are some white spots on the surface of fibers, which should be Fe_3_O_4_ nanoparticles doped into the fibers.

**Figure 5:**
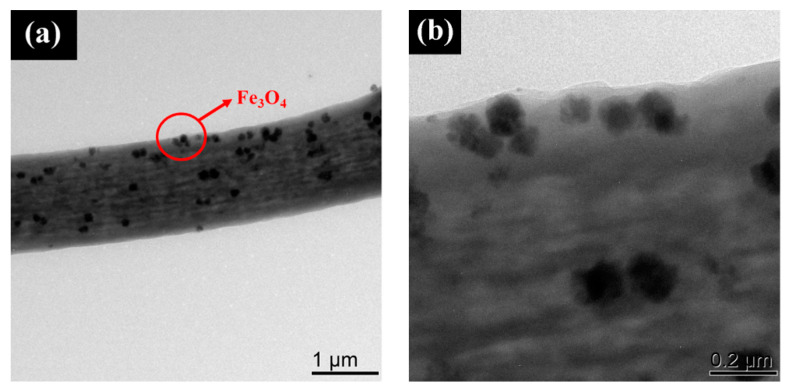
TEM images of sample 20Fe40Tb: (**a**) low resolution image; (**b**) high resolution image. Lina Liu et al.

**Figure 6:**
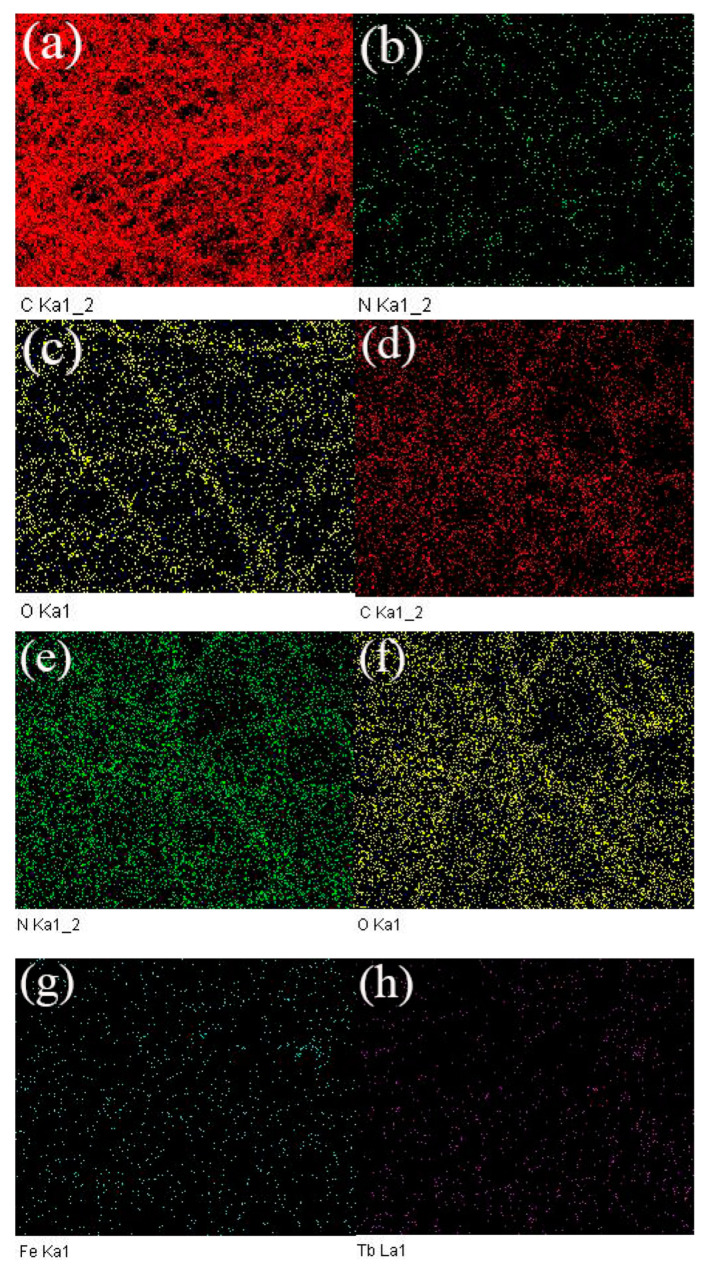
SEM-EDAX mapping of (**a**–**c**) pure PS microfibers and (**d**–**h**) sample 20Fe40Tb. Lina Liu et al.

In order to further characterize the successful doping of Fe_3_O_4_ nanoparticles into the composite microfibers, TEM images of sample 20Fe40Tb are given in Figure 5. We could clearly see the Fe_3_O_4_ nanoparticles in the composite microfibers. The SEM-EDAX mapping of pure PS microfibers and sample 20Fe40Tb are shown in Figure 6, in which the distributions of C, N, O, Fe, and Tb elements were shown. The outline of the microfibers could be roughly seen from the element distribution of C, N, and O. Fe and Tb elements were almost evenly distributed in sample 20Fe40Tb, indicating the uniform distributions of Fe_3_O_4_ nanoparticles and Tb(acac)_3_phen complexes in the bifunctional fibers.

**Figure 7:**
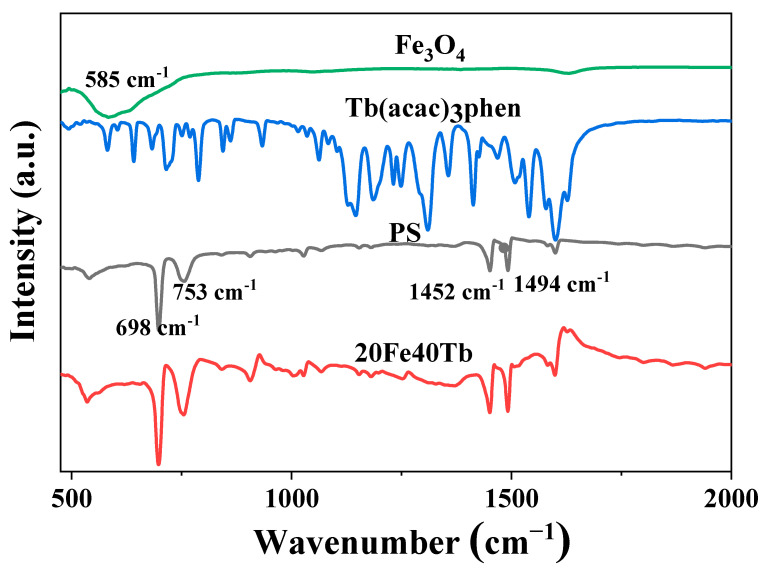
FTIR spectra of Tb(acac)_3_phen complexes, PS microfibers, Fe_3_O_4_ nanoparticles, and sample 20Fe40Tb. Lina Liu et al.

The FTIR spectra of pure Tb(acac)_3_phen complexes, PS fibers, Fe_3_O_4_ nanoparticles, and sample 20Fe40Tb are shown in Figure 7. The characteristic absorption band of the Fe-O bond was at 585 nm in Fe_3_O_4_ nanoparticles. The IR spectra of the bifunctional microfibers were similar to that of pure PS fibers, and no characteristic absorption peaks of the terbium complexes and Fe_3_O_4_ nanoparticles can be seen, indicating that most of the Tb(acac)_3_phen complexes and Fe_3_O_4_ nanoparticles in the microfibers were capped in the PS matrix [35,36]. For pure PS and bifunctional microfibers, the absorption peaks at 698, 753, 1452, and 1494 cm^−1^ were assigned to vibration absorption of benzene ring. These peaks maintained the same positions for the PS and bifunctional microfibers, indicating that Tb(acac)_3_phen complexes and Fe_3_O_4_ nanoparticles had little chemical interaction with PS and only physically mixed with it [35].

### 3.2. Photoluminescence Properties of Bifunctional Microfibers

**Figure 8:**
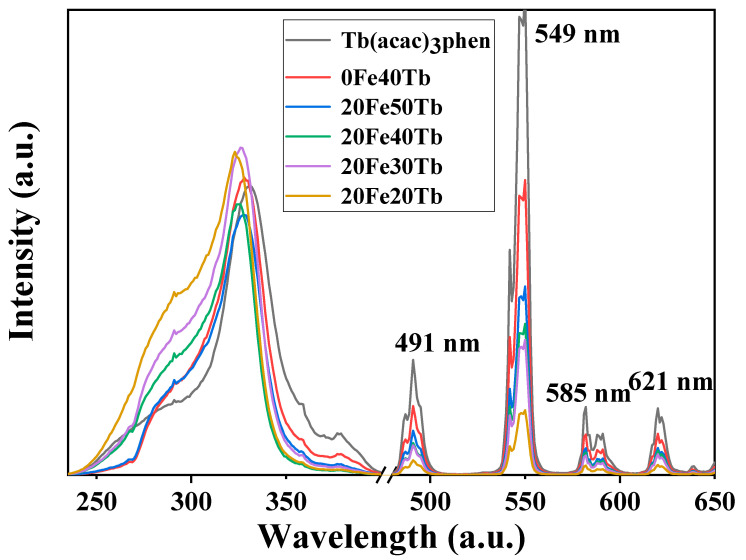
Excitation (*λ*_em_ = 550 nm) and emission spectra (*λ*_ex_ = 326 nm) of pure Tb(acac)_3_phen complexes and samples 0Fe40Tb, 20Fe50Tb, 20Fe40Tb, 20Fe30Tb, and 20Fe20Tb. Lina Liu et al.

The photoluminescence properties of samples with different doping concentrations of Tb(acac)_3_phen complexes were studied and compared them with pure terbium complexes and the bifunctional microfibers without Fe_3_O_4_ nanoparticles. Figure 8 shows the excitation and emission spectra of different samples. It could be seen from the excitation spectra that a broad excitation band ranging from 200 to 400 nm existed in all the excitation spectra, which corresponded to the π-π* electron transition of ligands [32]. The excitation peaks of the bifunctional fibers and the pure Tb(acac)_3_phen complexes did not completely overlap, and the excitation peaks of the bifunctional fibers blue shifted a small amount, which may have been caused by the change in the environment around the ligand [32]. The emission spectra indicated that all samples exhibited characteristic emission of terbium ion, and the main peak position was 549 nm, corresponding to the ^5^D_4_-^7^F_5_ transition of Tb^3+^. The shape and position of the emission spectra of all samples were similar, but the intensities were significantly different. The pure terbium complexes had the strongest emission spectrum, followed by the bifunctional fibers without the addition of Fe_3_O_4_ nanoparticles. Both ultraviolet and visible light can be absorbed by black Fe_3_O_4_ nanoparticles [20], indicating weaker emission spectra of the bifunctional fibers with the addition of Fe_3_O_4_ nanoparticles. When the doping ratios of the Fe_3_O_4_ nanoparticles were the same, the intensities of emission spectra gradually increased when adding more Tb(acac)_3_phen complexes.

In order to further investigate the photoluminescence properties of different samples, the relative luminescence intensities of samples 0Fe40Tb, 20Fe40Tb, and the pure Tb(acac)_3_phen complexes were calculated and are listed in Table 2. By comparing the intensities and concentration of different samples, we can see that the luminescence values of the unit mass of the Tb(acac)_3_phen complexes in samples 0Fe40Tb and 20Fe40Tb were stronger than that in the pure Tb(acac)_3_phen complexes, which meant that the outer luminescence efficiency values in samples 0Fe40Tb and 20Fe40Tb were improved [32]. Owing to the strong absorption of light by Fe_3_O_4_ nanoparticles, the luminescence of the unit mass of Tb(acac)_3_phen complexes in sample 20Fe40Tb was weaker than that in sample 0Fe40Tb but was still stronger than that in the pure Tb(acac)_3_phen complexes. In the pure complexes, a large part of excited-state energy was transferred into vibration transition energy because of strong electron–phonon coupling. When the Tb complexes were incorporated into the PS matrix, vibration transition was suppressed by PS. Therefore, more energy was transferred to Tb^3+^, leading to the improvement of photoluminescence.

**Figure 9:**
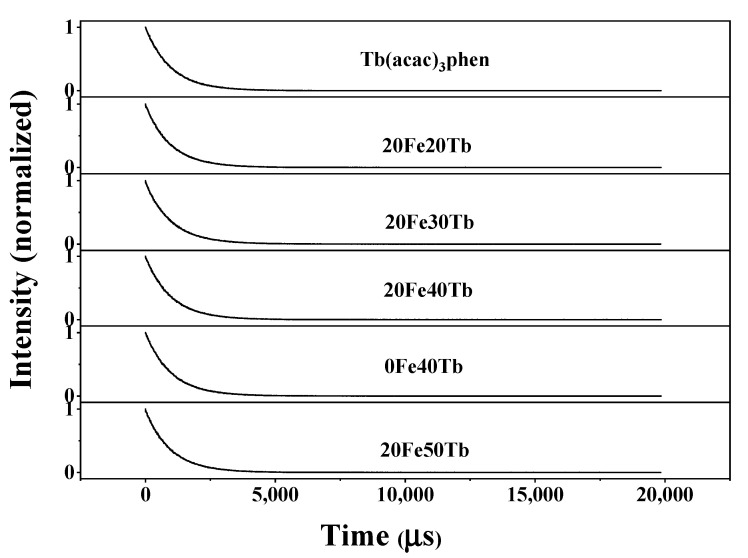
Fluorescence decay dynamics of the ^5^D_4_-^7^F_5_ transitions (*λ*_ex_ = 305 nm) in samples 20Fe50Tb, 0Fe40Tb, 20Fe40Tb, 20Fe30Tb, 20Fe20Tb, and pure Tb(acac)_3_phen complexes (room temperature). Lina Liu et al.

Figure 9 shows the fluorescence decay curves of ^5^D_4_-^7^F_5_ emissions for Tb^3+^ under 305 nm excitation (room temperature). The ^5^D_4_-^7^F_5_ emissions decay exponentially in all samples. The exponential lifetimes for the ^5^D_4_ states are obtained to be 975 µs in the pure Tb(acac)_3_phen complexes, 965 µs in sample 20Fe20Tb, 989 µs in sample 20Fe30Tb, 977 µs in sample 20Fe40Tb, 984 µs in sample 0Fe40Tb, and 974 µs in sample 20Fe50Tb. The fluorescence lifetimes for the ^5^D_4_ state were almost the same in all samples. This phenomenon was contrary to previous references [32,35,37], which may have been caused by the large doping proportions of the terbium complexes in the bifunctional fibers.

**Figure 10:**
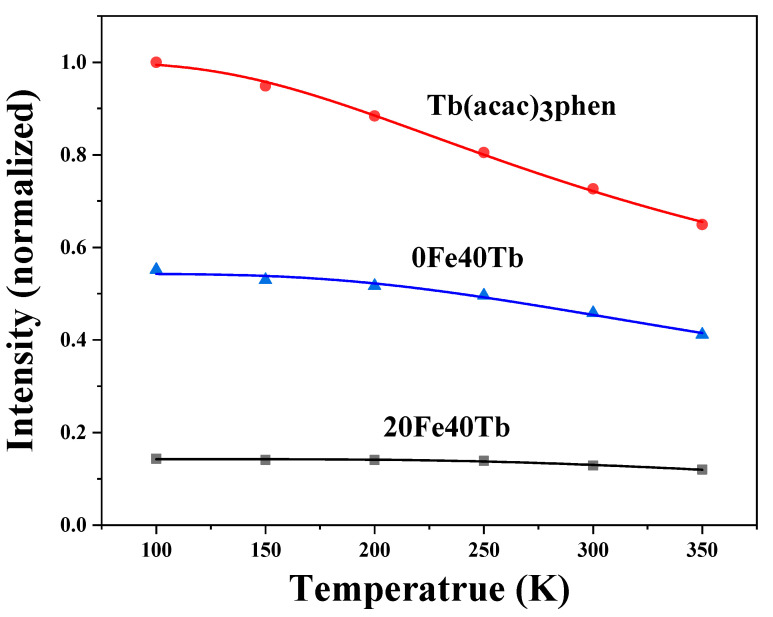
Dependence of emission intensity of the ^5^D_4_-^7^F_5_ transitions on temperature in samples 0Fe40Tb, 20Fe40Tb, and pure Tb(acac)_3_phen complexes. Lina Liu et al.

In order to compare the thermal stability of the photoluminescence of the pure Tb complexes and the Tb complexes in PS fibers, the temperature dependence of the photoluminescence intensities of the pure Tb complexes and the samples 0Fe40Tb and 20Fe40Tb were measured under 326 nm excitation and in a temperature range of 100–350 K. The temperature-dependent emission intensities of different samples are shown in Figure 10. For all samples, the ^5^D_4_-^7^F_5_ emission intensity of Tb^3+^ decreased monotonically with increasing temperature in the 100–350 K range. Compared with the pure Tb complexes, the emission intensities of ^5^D_4_-^7^F_5_ of Tb^3+^ in samples 0Fe40Tb and 20Fe40Tb decreased more slowly. As a function of temperature, the emission intensity was well fitted by the thermal activation equation, which could be written as [38]

$$I\left(T\right)=\frac{I_{0}}{1+\alpha e^{-E_{A}/K_{B}T}}$$

where $E_{A}$ is the activation energy of the thermal quenching process, $\kappa_{Β}$ is Boltzmann’s constant, α is the proportional coefficient, $I_{0}$ is the emission intensity at 0 K, and *T* is the absolute temperature. By fitting the experimental data using the above Equation, the thermal activation energy $E_{A}$ values of the pure terbium complexes and the samples 0Fe40Tb and 20Fe40Tb were 56.5, 82.9, and 125 meV, respectively. Compared with pure terbium complexes, the thermal activation energy of the bifunctional fibers was significantly improved, which indicated that the photoluminescence temperature stability was higher than that of pure Tb complexes [36]. In the bifunctional fibers, the PS matrix limited the ligand vibrations and reduced the non-radiative transition caused by ligand vibrations, resulting the improved luminescence thermal stability of the terbium complexes in the bifunctional fibers.

### 3.3. Magnetic Properties of Bifunctional Microfibers

**Figure 11:**
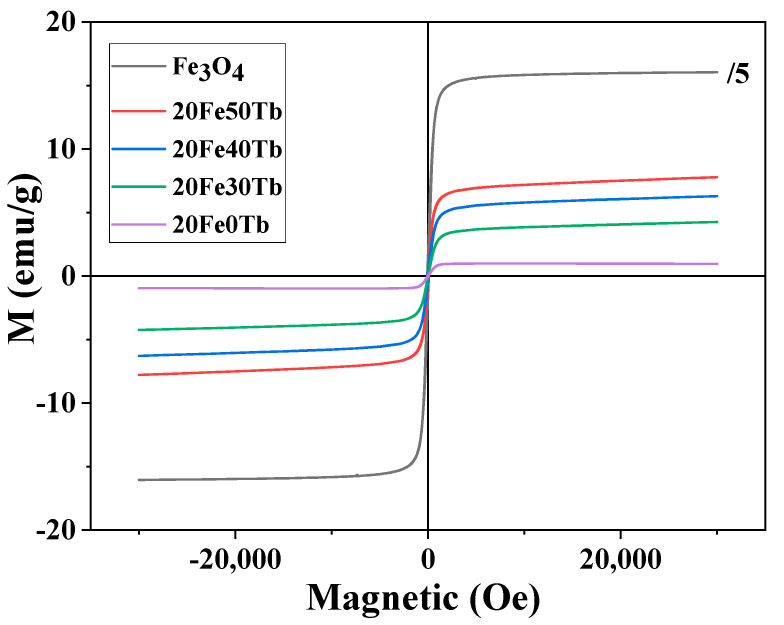
The magnetic hysteresis loops of samples 20Fe50Tb, 20Fe40Tb, 20Fe30Tb, 20Fe0Tb, and pure Fe_3_O_4_ nanoparticles. Lina Liu et al.

In order to characterize the magnetic properties of bifunctional fibers, Hysteresis loops of different samples are shown in Figure 11. The Y-axis value of pure Fe_3_O_4_ nanoparticles was contracted to a fifth of its original value. As can be seen from Figure 11, all samples were superparamagnetic. When the external magnetic field was added, the samples were magnetic, and the magnetism of the samples disappeared when the external magnetic field was removed, which made it possible for them to be applied in the biomedical field [39,40]. The saturation magnetization values of the Fe_3_O_4_ nanoparticles, samples 20Fe0Tb, 20Fe30Tb, 20Fe40Tb, and 20Fe50Tb, were 80.0, 1.1, 4.1, 6.1, and 7.4 emu/g, respectively. The reduced proportions of the Fe_3_O_4_ in bifunctional microfibers lead to smaller saturation magnetization properties. Interestingly, the saturation magnetization of the bifunctional microfibers with terbium complex doping was larger than that of bifunctional microfibers without terbium complex doping. Furthermore, the saturation magnetization of the bifunctional fibers gradually increased with the increase in the doping ratios of terbium complexes.

## 4. Conclusions

Fe_3_O_4_/Tb(acac)_3_phen/PS bifunctional microfibers with both magnetic and optical properties were prepared by electrospinning, and the structure, photoluminescence, and magnetic properties of the bifunctional microfibers were systematically studied. The experimental results indicated that Fe_3_O_4_ nanoparticles were successfully doped into PS microfibers. The characteristic emission peaks of Tb(acac)_3_phen complexes were observed in all bifunctional microfibers, which were located at 491, 549, 585, and 621 nm. The pure terbium complexes had the strongest emission spectrum, followed by the bifunctional fibers without the addition of Fe_3_O_4_ nanoparticles. The unit mass of the Tb(acac)_3_phen complexes in bifunctional microfibers was 1.2 times the luminescence intensity of that in pure Tb(acac)_3_phen complexes. The thermal activation energy $E_{A}$ of the pure terbium complexes and the bifunctional microfibers was 56.5 and 125 meV, indicating that the photoluminescence temperature stability was higher than that of pure Tb complexes. The saturation magnetization of the bifunctional fibers gradually increased with the increase in the doping ratios of terbium complexes. In our work, the optical and magnetic properties of Tb complexes and Fe_3_O_4_ nanoparticles were improved through combining, which made it possible for the bifunctional microfibers to be applied in biomedical and biochemical fields such as targeted drug delivery, magnetic resonance imaging, early diagnosis, and therapy of tumor cells.

## Figures and Tables

**Table 1:** The mass ratios of Tb(acac)_3_phen complexes and Fe_3_O_4_ nanoparticles to PS in different samples.

Sample	Mass Ratio of Tb(acac)_3_phen Complexes to PS (%)	Mass Ratio of Fe_3_O_4_ Nanoparticles to PS (%)
20Fe20Tb	20	20
20Fe30Tb	30	20
20Fe40Tb	40	20
20Fe50Tb	50	20
20Fe0Tb	0	20
0Fe40Tb	40	0
PS	0	0

**Table 2:** The content of Tb(acac)_3_phen complexes and relative emission intensities of samples 0Fe40Tb, 20Fe40Tb, and pure Tb(acac)_3_phen complexes.

Sample	Tb(acac)_3_phen	0Fe40Tb	20Fe40Tb
Content of Tb(acac)_3_phen (mass %)	100	28.3	24.8
relative intensities of ^5^D_4_-^7^F_5_	1	2.15	1.27

## Data Availability

Not applicable.
